# Investigations on Alkanediols as Alternative Preservatives in a Nonionic Hydrophilic Cream

**DOI:** 10.3390/pharmaceutics12111117

**Published:** 2020-11-20

**Authors:** Melanie Sigg, Rolf Daniels

**Affiliations:** Department of Pharmaceutical Technology, Eberhard Karls University, Auf der Morgenstelle 8, 72076 Tuebingen, Germany; melanie.sigg@uni-tuebingen.de

**Keywords:** alkanediols, preservation, amphiphilicity, rheology, DSC, X-ray

## Abstract

Alkanediols are often used as alternative antimicrobial preservatives for dermal formulations, e.g., hydrophilic creams. These substances show an antimicrobial effect due to their amphiphilic structure. At the same time, their amphiphilic behavior enables various interactions with the cream base itself. Therefore, the effect of four different alkanediols, namely 1,2-pentanediol, 2-methyl-2,4-pentanediol (hexylene glycol), 1,2-hexanediol, and 1,2-octanediol on the physical stability of a nonionic hydrophilic cream was investigated. Further, the incorporation of the alkanediols into lamellar structures of the cream was evaluated using differential scanning calorimetry (DSC) and small-angle X-ray scattering (SAXS) measurements. The interaction with the mixed crystals of the cream was found to increase with raising alkyl chain length of the added alkanediol. As a result, consistency and stability of the cream are slightly impaired. A test for efficacy of antimicrobial preservation according to the European Pharmacopoeia (Ph.Eur.) revealed that the antimicrobial activity is directly linked to the length of the alkyl chain of the alkanediols. 2-Methyl-2,4-pentanediol differs from both findings. This compound has non-vicinal hydroxy groups which result in a reduced amphiphilicity compared to the others. Consequently, it has a smaller impact on the colloidal structure of the cream and shows a comparatively low antimicrobial activity.

## 1. Introduction

**Figure 1:**
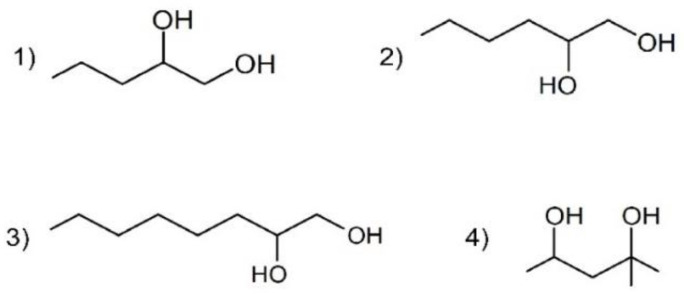
Chemical structures of the alkanediols used: (1) 1,2-pentanediol, (2) 1,2-hexanediol, (3) 1,2-octanediol, (4) 2-methyl-2,4-pentanediol.

Many commonly used antimicrobial preservatives in dermal formulations are increasingly under discussion, especially when they are used in pediatric formulations [1]. Moreover, some preservatives, e.g., parabens, are under debate due to their endocrine disrupting properties [2]. Hence, there is a growing need for alternative compounds for antimicrobial preservation in order to meet the microbiological quality acceptance criteria for cutaneous preparations. Alkanediols could be candidates for substituting conventional preservatives. The chemical structures of the alkanediols used in this study are presented in Figure 1.

1,2-Pentanediol, 1,2-hexanediol, and 1,2-octanediol are 1,2-alkanediols, having vicinal hydroxy groups on C1 and C2 positions. 2-Methyl-2,4-pentanediol, however, differs from this basic structure as it has two hydroxy groups on positions C2 and C4. The preservative action of these substances is ascribed to their amphiphilic properties which enable their integration into the cell membranes of micro-organisms [3]. Without having acidic or basic functional groups, alkanediols are antimicrobially effective over a wide pH range [4]. In addition to their antimicrobial effectiveness, these substances also exhibit moisturizing properties and are thus used as multifunctional excipients in cosmetic formulations [5]. 

**Figure 2:**
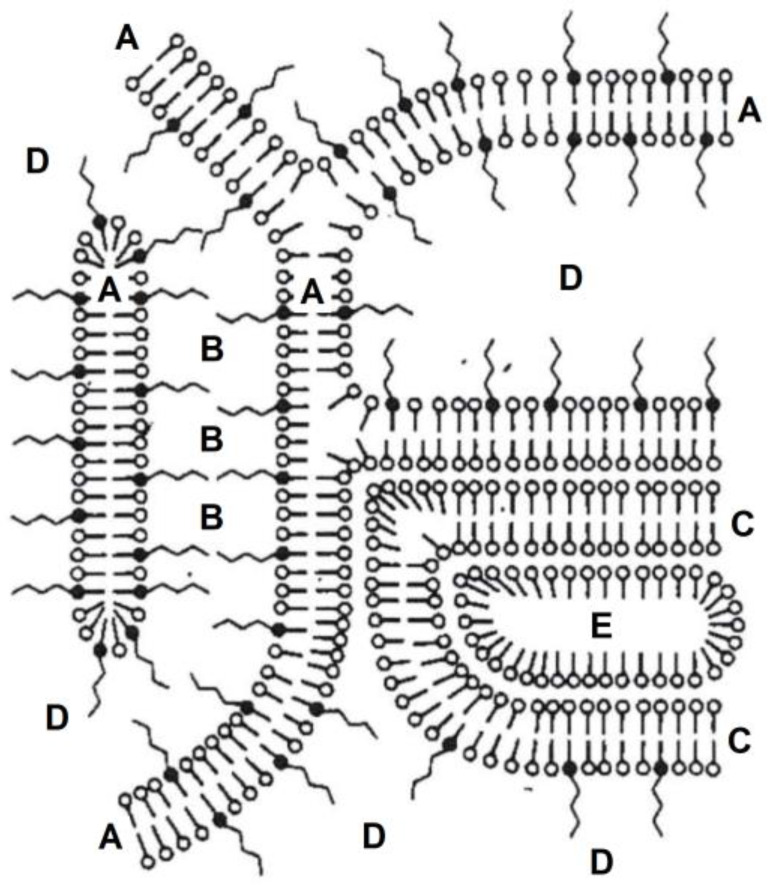
Model structure of the nonionic hydrophilic cream (NHC) according to the German Pharmacopeia (DAB) modified according to Junginger [7]: (A) mixed crystal of polysorbate 60 and cetolstearyl alcohol, (B) interlamellarly fixed water, (C) lipophilic gel phase, (D) bulk water, (E) lipophilic dispersed phase.

As a consequence of their amphiphilic structure, alkanediols also interact with the formulation itself [6]. To investigate this interaction, alkanediols were incorporated into a commonly used base in custom compounded creams. As an example, the nonionic hydrophilic cream (NHC) according to the German Pharmacopeia (DAB) was chosen. Its colloidal structure has been described in detail. Figure 2 schematically depicts its structure as proposed by Junginger [7]. The mixed crystal (A) is formed of polysorbate 60 and cetostearyl alcohol. Water can be accommodated and immobilized (B) within the lamellae of this mixed crystal. Both, the mixed crystal (A) and the immobilized water (B), together form the hydrophilic gel phase. The remainder of the cetostearyl alcohol which crystallizes as hemihydrate forms the lipophilic gel phase (C) which encloses the dispersed lipophilic phase (E). The bulk water phase (D) is immobilized within the coherent matrix structure of the two gel phases. 

It was the objective of the present investigations to analyze the effect of several alkanediols on the physical stability of NHC. Further, the incorporation of the alkanediols into the lamellar structures of the cream was evaluated using DSC and SAXS measurements. Finally, the efficacy of antimicrobial activity was tested. 

## 2. Materials and Methods 

### 2.1. Materials

1,2-Pentanediol, 2-methyl-2,4-pentanediol, and 1,2-hexanediol (Sigma-Aldrich Chemie GmbH, Taufkirchen, Germany), polysorbate 60 (Kolliphor PS 60, BASF SE, Ludwigshafen, Germany), cetostearyl alcohol (Kolliwax CSA, BASF SE, Ludwigshafen, Germany), glycerol 85% (Dr. Willmar Schwabe GmbH & Co. KG, Karlsruhe, Germany), white soft paraffin (Sasol Performance Chemicals, Hamburg, Germany), potassium sorbate (Caesar & Loretz GmbH, Hilden, Germany), citric acid (Jungbunzlauer Suisse AG, Basel, Switzerland). 1,2-Octanediol (dermosoft Octiol) was kindly donated by Evonik Dr. Straetmans GmbH, Hamburg, Germany.

### 2.2. Preparation of Test Formulations

The nonionic hydrophilic cream DAB was prepared according to the German Pharmacopoeia [8]. It has the following composition: polysorbate 60 5% (*w/w*), cetostearyl alcohol 10% (*w/w*), glycerol 85% 10% (*w/w*), white soft paraffin 25% (*w/w*), purified water 50% (*w/w*).

For antimicrobial preservation, either (i) no alkanediol, (ii) 5% 2-methyl-2,4-pentanediol, (iii) 5% 1,2-pentanediol, (iv) 3% 1,2-hexanediol, or (v) 1% 1,2-octanediol related to the whole formulation was added to the hydrophilic phase at 70 °C. These amounts correspond to the highest application concentrations found in literature [9,10,11]. The quantity of alkanediol was deducted from the amount of water. Further, three formulations were prepared using sorbic acid. Formulation (is) is the commonly used cream base which is preserved with solely 0.1% sorbic acid. Formulations (iis) and (iiis) are creams which were preserved with either 2-methyl-2,4-pentanediol or 1,2-pentanediol complemented with 0.05% sorbic acid. All manufactured formulations are summarized in Table 1.

After manual premixing, the formulations were finally homogenized using an Unguator 2100 mixing system (Gako International GmbH, Scheßlitz, Germany) with increasing rotation speed from 375 to 1900 rpm within 224 s.

### 2.3. Stability Study

After preparation, the creams were filled in airtight glass vials and stored for 6 months at room temperature. The stored samples were characterized by oscillation rheometry, polarization microscopy, and a centrifugation test after 1, 7, 14, 28 days, 3, and 6 months.

### 2.4. Rheological Measurements

Oscillation measurements were performed by means of a Physica MCR501 rheometer (Anton Paar, Graz, Austria) equipped with a plate-plate geometry (diameter: 25 mm; gap size: 0.2 mm). The temperature was set to 23 °C. The measuring cycle is displayed in Table 2. 

The storage modulus G’ in the linear viscoelastic region was determined and interpreted as a measure of the gel strength. Furthermore, the flow point was determined from an amplitude sweep as the shear stress at the cross over point (G’ = G’’). All measurements were performed in triplicate.

### 2.5. Polarization Microscope

Undiluted formulations were applied thinly to a glass microscope slide and covered with a coverslip. An Axio Imager Z1 microscope (Carl Zeiss, Jena, Germany) with crossed polarizers and a ¼ λ-plate was used to capture the polarization microscopic images of the samples at a magnification of 40×. 

### 2.6. Centrifugation Test

**Figure 3:**
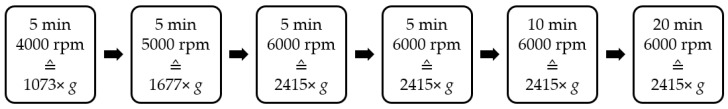
Diagram of the centrifugation test to determine the water holding capacity.

A centrifugation test was performed to determine the water holding capacity of the preparations. For this purpose, 1.25 g of each formulation (i), (ii), (iii), (iv), and (v) was weighed into a centrifugation tube and centrifuged in an Eppendorf MiniSpin Centrifuge (Eppendorf AG, Hamburg, Germany) according to the scheme indicated (Figure 3). After each centrifugation step, the formulations were visually examined with respect to water separation. If no phase separation occurred, the test was continued at the next stage.

### 2.7. Efficacy of Antimicrobial Preservation

The efficacy of antimicrobial preservation for formulations (is), (ii), (iis), (iii), (iiis), (iv), and (v) was tested according to the European Pharmacopoeia (Ph.Eur.) 5.1.3. [12]. In brief, after ensuring sterility at the beginning, the samples were inoculated each with a suspension of one of the test organisms (*Pseudomonas aeruginosa* ATCC 9027, *Staphylococcus aureus* ATCC 6538, *Candida albicans* ATCC 10231, and *Aspergillus brasiliensis* ATCC 16404 as described in the Ph. Eur. supplemented by *Eschericha coli* ATCC 8739). The microbial count of the suspension of inoculum was adjusted to yield 10^5^–10^6^ colony forming units (CFU) per gram after inoculation. The inoculated products were stored at 20–25 °C, protected from light. The total microbial count was determined 2 and 28 days after inoculation using the plate count method according to Ph. Eur. 2.6.12 [12]. Additionally, the count of the viable microorganisms was carried out after 7 and 14 days if the previous measurements resulted in total microbial count values of ≥10. Otherwise, it was assumed that the values are <10. To evaluate the antimicrobial activity, the log_10_ reduction in the number of viable micro-organisms against the value obtained for the inoculum was calculated and compared with the criteria for evaluation of antimicrobial activity according to Ph. Eur., as described in Table 3.

### 2.8. Preparation of Mixed Crystals for DSC and SAXS Measurements

The mixed crystal consists of polysorbate 60, cetostearyl alcohol, water and, if applicable, an alkanediol. For the preparation, polysorbate 60 and cetostearyl alcohol were melted at 70 °C. After heating water to the same temperature, the alkanediol was added and manually incorporated into the mixture of polysorbate 60 and cetostearyl alcohol. The mixed crystal was cooled to room temperature while stirring. The ratio of the incorporated alkanediol to the mixed crystal was the same as in the NHC prepared for the stability study.

### 2.9. Differential Scanning Calorimetry (DSC)

The melting points of the individual substances and the mixed crystals were determined by thermoanalytical measurement using a DSC 820 (Mettler Toledo GmbH, Gießen, Germany). About 20 mg of the sample were accurately weighed into a 40 µL aluminum crucible and cold sealed. The samples were cooled to −50 °C (for 1,2-hexanediol and 1,2-octanediol) or to −100 °C (for 1,2-pentanediol and 2-methyl-2,4-pentanediol), kept at this temperature for 2 min, and then heated to 80 °C, where they were again kept isothermally for 2 min. This temperature cycle was repeated a second time. The heating and cooling rate was 5 K/min, respectively. An empty crucible was used as reference. The second heating curve was evaluated. For presentation in the graph, all curves were standardized to the sample weight.

The limit of detection of the method for the samples containing 2-methyl-2,4-pentanediol, 1,2-pentanediol, and 1,2-hexanediol was determined by examining a sample containing the alkanediol, polysorbate 60, and cetostearyl alcohol in the same ratio as in the mixed crystal. No water was added. For determining the detection limit of the samples containing 1,2-octanediol, polysorbate 60 was excluded because its melting temperature is similar to that of 1,2-octanediol. In these curves a separate melting peak of the alkanediol was expected if the amount exceeded the limit of detection.

### 2.10. Small-Angle X-ray Diffraction Measurements

X-ray measurements were performed with a small-angle X-ray diffraction system SAXSess mc² equipped with a CCD (charge-coupled device) detector (Anton Paar GmbH, Graz, Austria). A copper anode with a CuKα line wavelength of 0.154 nm served as X-ray source. The samples were measured in a quartz capillary sample holder at 20 °C. Per measuring series 25 runs with an exposure time of 30 s were averaged.

The X-ray diffraction measurements were analyzed based on Bragg’s equation

2 d sinθ = n λ(1)

where in d represents the distance of the lattice layers of the measured crystals, θ denotes the angle of the incident X-rays referred to the planes (thus θ corresponds to half the angle between the incident and the reflected X-ray beams), λ is the wavelength of the X-ray beam, and n is a positive integer. 

**Figure 4:**
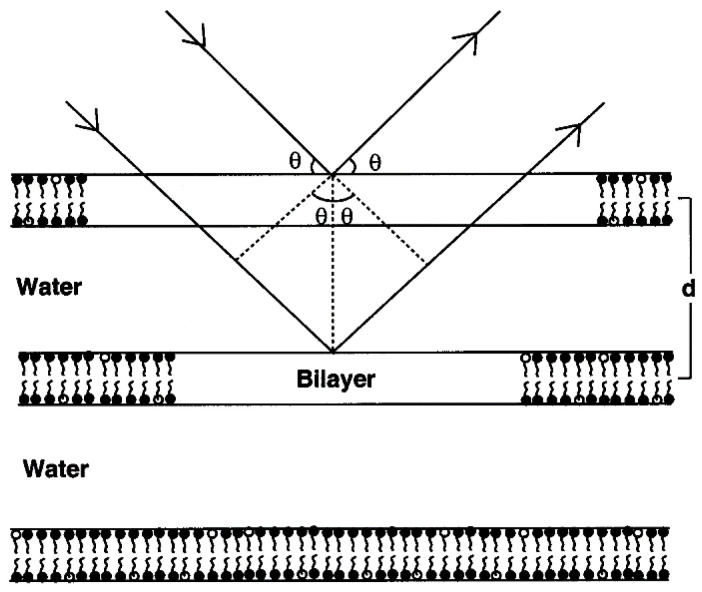
Schematic diagram of X-ray diffraction from bilayers reproduced with permission from G.M Eccleston, International Journal of Pharmaceutics; published by Elsevier, 2000 [13].

In a crystal, d represents the interplanar distance of lattice planes. However, in the mixed crystals examined in this study d denotes the distance between the bilayers of mixed crystals as displayed in Figure 4. Thus, in our study, d describes the lamellar spacing.

As can be recognized from Bragg’s equation, θ is inversely proportional to the distance of the lamellar spacing as long as sinθ can be approximated by θ.

## 3. Results and Discussion

### 3.1. Influence of Alkanediols on the Storage Stability

**Figure 5:**
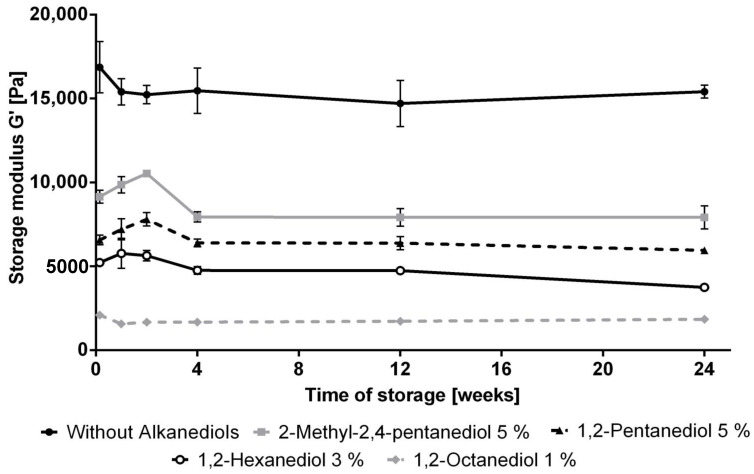
Influence of alkanediols on the storage modulus G’, *n* = 3, mean ± standard deviation.

All produced NHCs were white semisolids. Within the storage time of 6 months all formulations showed no phase separation during all six stages of the centrifugation test. Additionally, the consistency of all creams remained unchanged. This finding is supported by the corresponding oscillation measurements (Figure 5). Figure 5 presents the storage moduli G’ in the linear viscoelastic region of the prepared creams containing the different alkanediols during 6 months storage at room temperature. During the first 4 weeks, some of the creams show small changes in G’ which are typical for that kind of formulation due to rearrangement of colloidal structures after intense shearing during preparation. After 1 month storage, all G’ values remained nearly constant which is a clear indication that the consistency did not change essentially during storage. 

However, the consistency of the creams depends clearly on the type of the added alkanediol. Their gel strength decreases with increasing chain length of the alkanediol.

The most pronounced effect was observed for the storage modulus of the NHC supplemented with 1,2-octanediol which possesses the longest alkyl chain of the investigated alkanediols, followed by the creams containing 1,2-hexanediol and 1,2-pentanediol. 2-Methyl-2,4-pentanediol differs from this sequence. Preparations containing 2-methyl-2,4-pentanediol showed the highest storage modulus of all NHCs with the addition of alkanediols. This deviation is presumably a consequence of the different, non-vicinal positions of the hydroxy groups of this substance.

**Figure 6:**
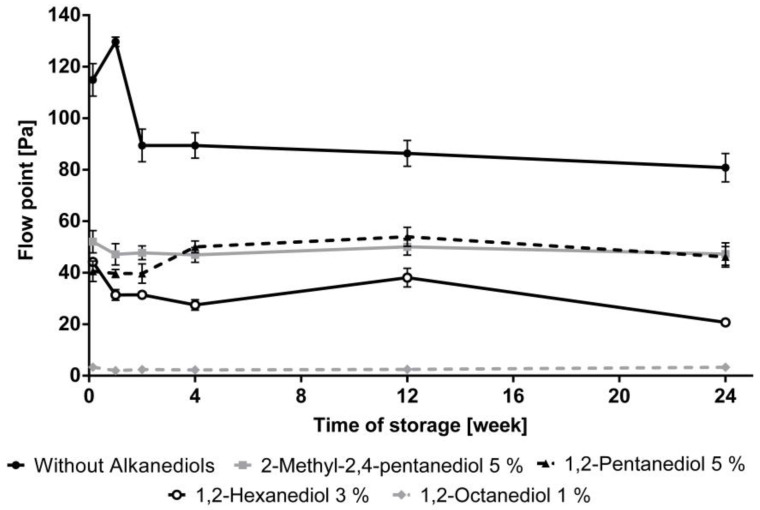
Influence of alkanediols on the flow point, *n* = 3, mean ± standard deviation.

The flow points of the different creams during storage are displayed in Figure 6. The NHC without the addition of alkanediols demonstrated the highest flow point. The formulations containing 1,2-pentanediol and 2-methyl-2,4-pentanediol showed similar flow points, whereas its value for the cream with 1,2-hexandiol was clearly reduced. 1,2-Octanediol has the highest impact on the flow point of the tested creams. It is worth to mention, that although the addition of 1,2-octanediol yielded very low flow points, the samples did not freely flow under the influence of gravity from the storage container.

**Figure 7:**
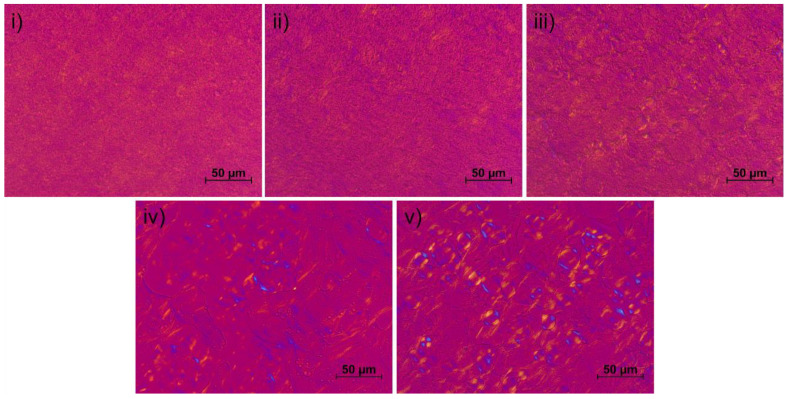
Polarization microscopic images of the formulations stored over 6 months: (**i**) pure NHC, (**ii**) 2-methyl-2,4-pentanediol 5%, (**iii**) 1,2-pentanediol 5%, (**iv**) 1,2-hexanediol 5%, (**v**) 1,2-octanediol 1%.

Figure 7 represents the effect of the different alkanediols on the microscopic appearance of the NHC after 6 months storage. The pure NHC (i) and the NHC with the addition of 5% of 2-methyl-2,4-pentanediol (ii) show no visible textures. This indicates that the lamellar structures are small compared to the resolution of the polarization microscope. Further, supplementing the NHC with 1,2-pentanediol (iii) does not significantly alter the appearance compared to the pure NHC.

In contrary, the formulations with longer-chained alkanediols show clearly visible Maltese crosses indicating the presence of a lamellar phase in the cream [14]. Obviously, these longer-chain amphiphilic molecules provoked a rearrangement in the liquid crystalline structures of the creams which led to the formation of larger and thus detectable crystals.

### 3.2. Incorporation of Alkanediols into Mixed Crystals

DSC and SAXS measurements were performed to further elucidate the impact of the alkanediols on the colloidal structure of the cream which was indicated by rheometry and polarized light microscopy. To this end, mixed crystals consisting of polysorbate 60, cetostearyl alcohol, and water were prepared without and with additional alkanediols.

#### 3.2.1. DSC Measurements

**Figure 8:**
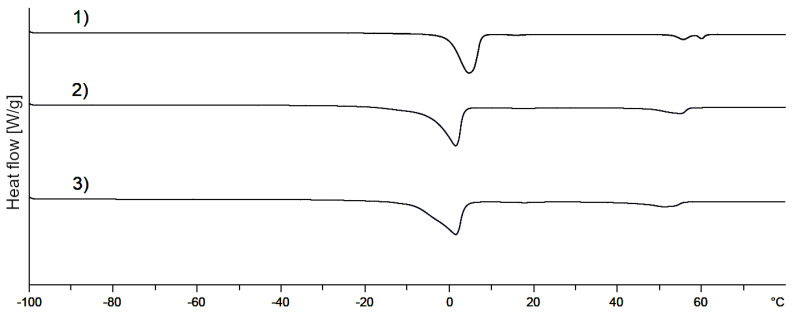
DSC thermograms of (1) mixed crystal without alkanediols; (2) mixed crystal: 2-methyl-2,4-pentanediol, standard concentration; (3) mixed crystal: 1,2-pentanediol, standard concentration.

DSC thermograms of mixed crystals without and with 1,2-pentanediol and 2-methyl-2,4-pentanediol are shown in Figure 8. The addition of these two alkanediols (curves 2 and 3) seems to change the thermal behavior only marginally compared to plain NHC (curve 1). The melting point of the mixed crystal with additional alkanediols is slightly shifted to lower temperatures. However, it must be taken into consideration that the concentration might be too low in order to allow the detection of a separate melting peak if present. Therefore, it cannot finally be decided from the DSC measurements whether these two alkanediols are integrated into the mixed crystal or not.

**Figure 9:**
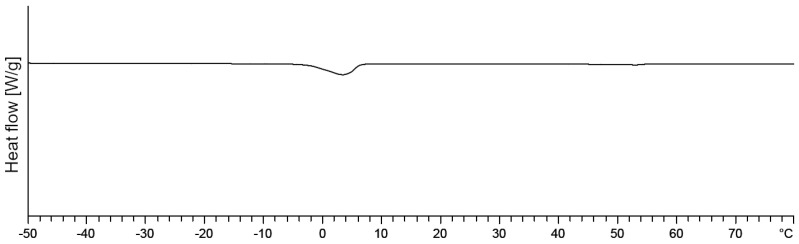
DSC thermogram of a mixed crystal containing 1,2-hexanediol, standard concentration.

The thermogram of the mixed crystal with 1,2-hexandiol shows only one melting peak as displayed in Figure 9. As the concentration of 1,2-hexanediol was clearly above its limit of detection, this finding indicates a complete incorporation of 1,2-hexanediol into the mixed crystal.

**Figure 10:**
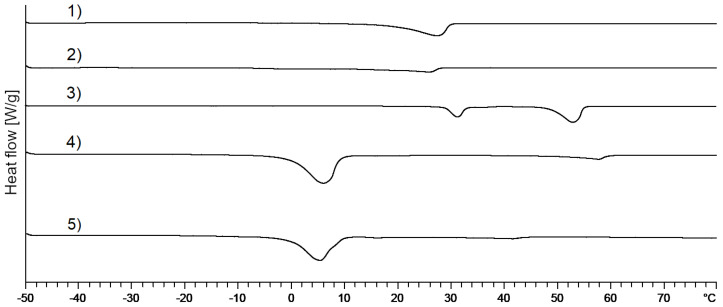
DSC thermograms of (1) 1,2-octanediol; (2) polysorbate 60; (3) cetostearyl alcohol; (4) mixed crystal: 1,2-octanediol, standard concentration; (5) mixed crystal: 1,2-octanediol, 10-fold concentration.

Figure 10 depicts the thermograms of mixed crystals supplemented with 1,2-octanediol. Curves 1–3 show the melting behavior of the pure substances 1,2-octanediol (curve 1), polysorbate 60 (curve 2), and cetostearyl alcohol (curve 3). The curve of the mixed crystal with 1,2-octandiol (curve 4) shows a singular endothermic melting peak at 0.11 °C indicating a complete integration of 1,2-octanediol into the lamellar mixed crystal. In order to further challenge this system, a mixed crystal with the 10-fold amount of 1,2-octanediol was prepared. As can be seen form curve 5, also at this overdosed concentration only one endothermal event and no separate melting peak of 1,2-octandiol could be detected. This implies that 1,2-octanediol is fully incorporated into the mixed crystal at least in the concentration range from 0 to 10% including its standard concentration of 1%. 

**Figure 11:**
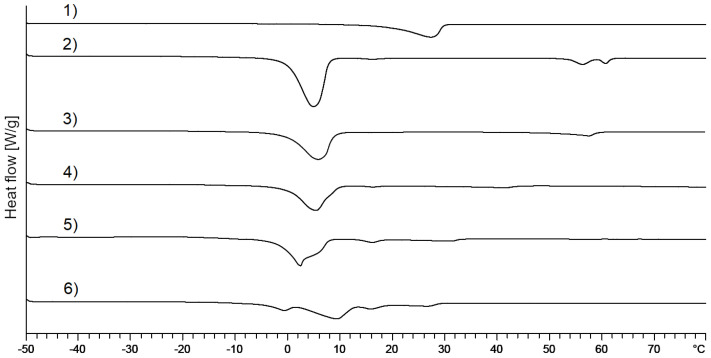
DSC thermograms of (1) 1,2-octanediol; (2) mixed crystal without alkanediols; (3) mixed crystal: 1,2-octanediol, standard concentration; (4) mixed crystal: 1,2-octanediol, 10-fold concentration; (5) mixed crystal: 1,2-octanediol, 20-fold concentration; (6) mixed crystal: 1,2-octandiol, 30-fold concentration.

As the 1,2-octanediol concentration is further increased (Figure 11: curve 5: 20%; curve 6: 30%), the melting peaks begin to widen with increasing concentration of 1,2-octanediol. This also suggests an interaction of 1,2-octanediol with the mixed crystal leading to a less ordered structure if the content of 1,2-octanediol is augmented. 

#### 3.2.2. SAXS Diffraction

**Figure 12:**
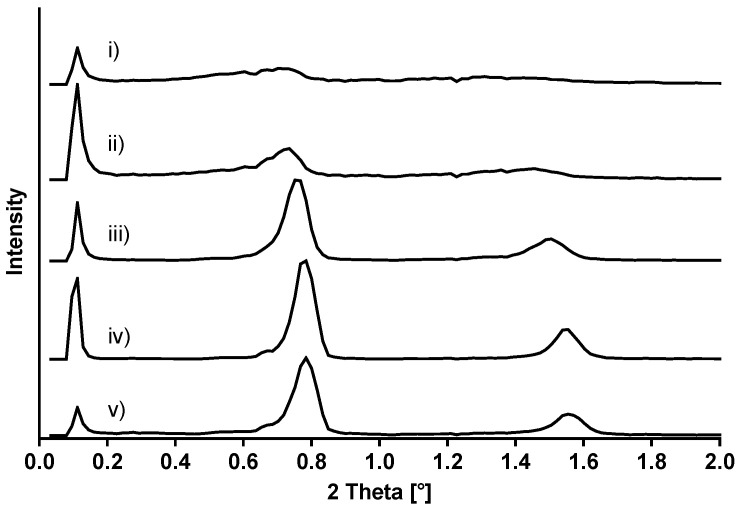
SAXS diffraction patterns of mixed crystals of (i) cetostearyl alcohol, polysorbate 60 and water in the presence of (ii) 2-methyl-2,4-pentanediol, (iii) 1,2-pentanediol, (iv) 1,2-hexanediol, (v) 1,2-octanediol.

In order to determine the interlamellar spacing of the various mixed crystals in the absence and presence of alkanediols, SAXS diffraction measurements were performed (Figure 12). The reflected intensity is plotted against the scattering angle 2θ, the angle between incident and diffracted X-ray beam. The corresponding lattice layer distances are summarized in Table 4. The order of magnitude of the layer lattice distances is defined by cetostearyl alcohol [13,15,16,17].

All samples show a peak in the SAXS diffraction pattern at 2θ = 0.11° (d = 78.52 nm) which represents the long-range order of the mixed crystals. In contrast, the peaks representing the near-order region are clearly affected by the addition of alkanediols. The spectrum of the mixed crystal without any alkanediol (i) shows no distinct, sharp peak. The addition of 2-methyl-2,4-pentanediol to the mixed crystal (ii) only leads to a small change in the diffraction pattern characterized by two peaks which can be clearly identified. First-order reflections spectra of high intensity were obtained when adding 1,2-alkanediols (iii)–(v) to the mixed crystals. This is a clear indication that the 1,2-alkandiols interact with the lamellar structure of cetostearyl alcohol and polysorbate 60 provoking a rearrangement leading to more ordered and regularly organized structures [14]. This finding is consistent with the results of the polarization microscopic images which indicate a more pronounced lamellar phase when higher chained alkanediols are incorporated into the mixed crystals.

The ratio of the SAXS reflexes points directly to the lipid phase structure. If the ratio of the reflections is 1:2, 1:3, 1:4, etc., this is indicative of lamellar structures [14]. The plain cetostearyl alcohol and polysorbate 60 mixed crystals revealed a ratio of the angular distances of first and second order peaks of 1.86. This can be attributed to the fact that cetostearyl alcohol is a mixed compound which is not able to perfectly order in lamellae with a distinct spacing. The addition of alkanediols increases this ratio to values close to 2. This is not only a distinct sign of the existence of lamellar structures but also strengthens the hypothesis that the incorporation of the 1,2-alkandiol allows for a rearrangement of the fatty alcohol molecules.

Beside the peaks getting sharper and the lamellar structures getting more pronounced with addition of alkanediols, the scattering angle 2θ is shifted to higher values, as can be seen in Figure 12. The mixed crystal without an alkanediol reveals interlamellar spacings of 12.56 and 6.74 nm. Upon the addition of 1,2-alkandiols, the distance of the lattice layers of the mixed crystal decreases with increasing alkyl chain length of the 1,2-alkanediol, leading to layer distances of 11.25 and 5.68 nm in the presence of 1,2-hexandiol and 1,2-octandiol. This result suggests that the rigid crystals become more flexible upon the incorporation of the amphiphilic 1,2-alkanediols. This allows the fatty alcohol molecules to come more closely together and reduces thus the layer lattice distances. 

The reduction of lamellar spacing due to a rearrangement of the lipids can be a hint that less water is enclosed between the layers of the mixed crystal [13]. This shifts the ratio between interlamellarly fixed water and bulk water in direction of the bulk water. Hence, the gel structure of the nonionic cream has to immobilize a larger amount of bulk water [18]. If this is not possible, the reorganization of liquid crystalline and crystalline phases reduces the ability of the cream to immobilize water making the formulations less stable.

In contrast, 2-methyl-2,4-pentanediol showed only a small impact on the spacing of the layers leading to distances of 12.00 and 6.06 nm, respectively. Compared to the 1,2-alkanediols, the incorporation of 2-methyl-2,4-pentanediol into the mixed crystal is less favorable because it has the hydroxyl groups on the positions C2 and C4. This reduces its amphiphilicity and consequently, both, the peak intensity and the layer distance resemble the plain mixed crystal. 

### 3.3. Efficacy of Antimicrobial Preservation

To evaluate the preservative activity of the different alkanediols, a test for efficacy of antimicrobial preservation was performed. The results were evaluated according to the acceptance criteria specified in the Ph. Eur. [12]. According to Ph. Eur., the log_10_ reduction in the number of viable micro-organisms against the value obtained for the inoculum serves as the basis for the evaluation. Criterion A represents the usually required efficacy. If, in justified cases, criterion A cannot be met, e.g., if there is an increased risk of adverse effects, criterion B must be attained.

Table 5 summarizes the results of the test for efficacy of antimicrobial preservation. NHC conventionally preserved with 0.1% sorbic acid (is) served as a positive control. Formulations containing 5% 2-methyl-2,4-pentanediol (ii), 5% 1,2-pentanediol (iii), 3% 1,2-hexanediol (iv), and 1% 1,2-octanediol (v) were tested. For the creams with shorter chained alkanediols, formulations were analyzed containing additionally 0.05% sorbic acid, which is half the concentration typically used in dermal formulations (Table 5: formulations (iis) and (iiis)). These creams were tested to investigate whether the concentration of a commonly used preservative can be reduced in case the alkanediol alone does not have a sufficient preservative effect.

The formulations containing the recommended amount of 1,2-hexanediol (iv) and 1,2-octanediol (v) met the “A” criterion according to Ph. Eur. for all tested strains. This underlines that the increasing amphiphilicity of these compounds increases their antimicrobial activity even at the lower use concentrations.

On the contrary, the amphiphilic character of 1,2-pentanediol is less pronounced leading to partially impaired antimicrobial activity. Consequently,1,2-pentandiol achieved only criterion B for *Asp. brasiliensis*. However, this weak effect against the mold could be compensated by supplementing the cream with 0.05% sorbic acid.

The lowest antimicrobial activity was found for 2-methyl-2,4-pentanediol which failed both Ph. Eur. criteria for *Asp. brasiliensis*. As already discussed above, it is supposed that this is a consequence of the location of its hydroxy groups in positions 2 and 4 reducing the amphiphilicity and thus making an integration into the lipid bilayer of the cell membrane of the microorganism less favorable. However, even in this case supplementing the cream with 0.05% sorbic acid was sufficient to attain the “A” criterion for *Asp. brasiliensis*. Although it is not sufficiently effective on its own, 2-methyl-2,4-pentanediol can therefore be used to reduce the concentration of the commonly used preservative sorbic acid.

These results are completely in line with the proposed mechanism of antimicrobial activity of the alkanediols which is attributed to their amphiphilic structure. Hence, these substances can penetrate the lipid bilayer of the microbial cell membrane. As a result, substrate transport is inhibited, and vital cellular components leak. The disruptive effect increases as a consequence of the deeper penetration into the bilayer as a function of the chain length [19]. The surface activity of the alkanediols increases with increasing chain length of the alkyl chain (data not shown). 2-Methyl-2,4-pentanediol differs from this sequence due to its non-vicinal hydroxy groups. Consequently, its interfacial activity and its antimicrobial activity are markedly reduced.

## 4. Conclusions

In the present study, alkanediols were investigated as alternative preservatives for the nonionic hydrophilic cream DAB as a typical representative of a nonionic cream. The antimicrobial activity of these compounds is linked to their amphiphilic structure. The latter could be easily demonstrated by measuring the interfacial activity of these compounds. Their amphiphilic character enables on the one hand the interaction with cell membranes of microorganisms but allows on the other hand also to affect the colloidal structure of creams. The present study revealed that alkanediols change the colloidal structure of the NHC as a consequence of the incorporation of these amphiphilic substances into the mixed crystal of the cream. The detected decrease of the lamellar distances of the mixed crystal results in a diminished consistency of the creams. The observed reduction of the storage modulus G’ and the flow point was in line with the chain length of the added alkanediol and came along with an increasingly ordered mixed crystal which is responsible for the semi-solid behavior of the cream. The lamellar nature manifests in characteristic “Maltese cross” textures which become visible under a polarized microscope when the NHC is supplemented with 1,2-hexanediol or 1,2-octanediol. This means, the stability of the NHC is reduced by the incorporation of an alkanediol in its mixed crystal. However, as no visible phase separation was detected even during an extended centrifugation test, it can be concluded that the changes in the colloidal structure did practically not affect the ability of the lamellar gel network to immobilize the incorporated water.

Furthermore, alkanediols proved to be excellent alternatives for the antimicrobial preservation of the NHC, wherein the preservative activity raises with their alkyl chain length and could be directly linked to their amphiphilicity. All formulations containing 1,2-alkanediols attained mostly criterion “A” in the test for efficacy of antimicrobial preservation. 1,2-octanediol proved to have the highest activity, whereas 1,2-pentanediol was less efficient and achieved only criterion B for the test mold. 

The only alkanediol, that differs largely from the others in all addressed properties was 2-methyl-2,4-pentanediol which differs in its chemical structure from the other alkanediols as it has no vicinal hydroxy groups. It showed the lowest interfacial activity and had the lowest impact on both the stability and the preservation of the NHC. Its integration into the lamellar lipid structure of the cell membranes as well as the mixed crystal of the NHC is sterically hindered. 

In general, it can be concluded, that 1,2-alkanediols can be used as effective alternative preservatives in dermal formulations. Changes in the consistency of the preparation ultimately limit the use of 1,2-alkanediols. Besides, ongoing studies are aiming to clarify if such an alternative preservation affects release and skin penetration of drug substances. 

## Figures and Tables

**Table 1:** Overview of test formulations.

Formulation	Additive	Concentration (%)
(i)	-	-
(is)	Sorbic acid	0.1
(ii)	2-methyl-2,4-pentanediol	5
(iis)	2-methyl-2,4-pentanediol + sorbic acid	5 + 0.05
(iii)	1,2-pentanediol	5
(iiis)	1,2-pentanediol + sorbic acid	5 + 0.05
(iv)	1,2-hexanediol	3
(v)	1,2-octanediol	1

**Table 2:** Measurement parameters of the rheological investigations.

Phase	Condition	Duration (min)
Pre-shear	Shear rate = 5 s^−1^	1
Rest	-	2
Amplitude sweep	Logarithmically from 0.01% to 100%; 1 Hz	

**Table 3:** Criteria for evaluation of antimicrobial activity for preparations for cutaneous application according to Ph. Eur.

Microorganisms	Log_10_ Reduction				
	Criteria	2 Days	7 Days	14 Days	28 Days
**Bacteria**	A	2	3	-	NI
	B	-	-	3	NI
**Fungi**	A	-	-	2	NI
	B	-	-	1	NI

NI: no increase in number of viable micro-organisms compared to the previous reading.

**Table 4:** Lamellar distances of mixed crystals of cetostearyl alcohol, polysorbate 60, water and in the presence of different alkanediols.

Mixed Crystals	2θ (°)	D (nm)	Ratio
(i) in the absence of alkanediol	0.70	12.56	1.86
	1.31	6.74	
(ii) 2-methyl-2,4-pentanediol	0.74	12.00	1.98
	1.46	6.06	
(iii) 1,2-pentanediol	0.75	11.74	2.00
	1.51	5.86	
(iv) 1,2-hexanediol	0.78	11.25	1.98
	1.56	5.68	
(v) 1,2-octanediol	0.78	11.25	1.98
	1.56	5.68	

**Table 5:** Acceptance criteria according to the test for efficacy of antimicrobial preservation Ph. Eur. 5.1.3. achieved by the formulations: (is) NHC conventionally preserved with 0.1% sorbic acid, (ii) 5% 2-methyl-2,4-pentanediol, (iis) 5% 2-methyl-2,4-pentanediol + 0.05% sorbic acid, (iii) 5% 1,2-pentanediol, (iiis) 5% 1,2-pentanediol + 0.05% sorbic acid, (iv) 3% 1,2-hexanediol, (v) 1% 1,2-octanediol.

Formulation	(is)	(ii)	(iis)	(iii)	(iiis)	(iv)	(v)
*Staph. aureus*	A	A	A	A	A	A	A
*Ps. aeruginosa*	A	A	A	A	A	A	A
*E. coli*	A	A	A	A	A	A	A
*C. albicans*	A	A	A	A	A	A	A
*Asp. brasiliensis*	A	-	A	B	A	A	A
